# Relaxant effect of *Curcuma longa* on rat tracheal smooth muscle and its possible mechanisms

**DOI:** 10.1080/13880209.2017.1400079

**Published:** 2017-11-24

**Authors:** Bahman Emami, Farzaneh Shakeri, Vahideh Ghorani, Mohammad Hossein Boskabady

**Affiliations:** a Neurogenic Inflammation Research Centre, Mashhad University of Medical Sciences, Mashhad, Iran;; b Department of Physiology, School of Medicine, Mashhad University of Medical Sciences, Mashhad, Iran

**Keywords:** Beta adrenergic stimulation, muscarinic receptor inhibition, calcium channel blocking

## Abstract

**Context:** Turmeric is a spice obtained from the root of *Curcuma longa* L. (Zingiberaceae) with anti-aging, anticancer, anti-Alzheimer’s disease, antioxidant and other medicinal properties.

**Objective:** The relaxant effect of *C. longa* on rat tracheal smooth muscle and its possible mechanisms were investigated in this study.

**Materials and methods:** The relaxant effects of four cumulative concentrations of hydro-ethanol extract of *C. longa* (6.25, 12.5, 25, 50 mg/mL) were studied on tracheal smooth muscle precontracted by methacholine or KCl in non-incubated or incubated with different substances including propranolol, diltiazem, L-NAME, glibenclamide, atropine, chlorpheniramine, indomethacin and papaverine. The duration of the study was 84 days.

**Results:** In non-incubated tracheal smooth muscle, the extract of *C. longa* showed significant concentration-dependent relaxant effects (*p* < 0.001 for all concentrations on both KCl and methacholine-induced contraction). There was no significant difference in the relaxant effects between *C. longa* and theophylline in both methacholine and KCl-induced contraction conditions. In tissues incubated with propranolol, diltiazem, L-NAME and glibenclamide on methacholine-induced contraction and in tissues incubated with atropine, chlorpheniramine, indomethacin and papaverine on KCl-induced contraction, the extract also showed significant concentration-dependent relaxant effects (*p* < 0.001). EC_50_ values of *C. longa* between non-incubated (16.22 ± 0.62) and incubated tissues (atropine: 13.03 ± 0.55, chlorpheniramine: 12.94 ± 0.68, indomethacin: 14.80 ± 0.57 and papaverine: 16.16 ± 1.42) were not significantly different.

**Conclusions:** Tracheal smooth muscle relaxant effects of *C. longa*, were comparable to those of theophylline, which could be due to the presence of methylxanthines or its possible interaction with non-adrenergic non-cholinergic nervous system.

## Introduction

Turmeric, the common name of *Curcuma longa* L. (Zingiberaceae), is a perennial plant cultivated in India and other parts of Southeast Asia (Ammon and Wahl 1991). *C. longa* cultivation requires a hot (20–30 °C) and humid climate and great amounts of water (Esatbeyoglu et al. 2012). Turmeric contain; curcumin, demethoxycurcumin and bisdemethoxycurcumin, as well as volatile oils (i.e., tumerone, atlantone and zingiberone), sugars, proteins and resins (Singh et al. 2010). Curcumin (diferuloylmethane), the most important compound of the plant which is responsible for *C. longa* biological activities and is responsible for its vibrant yellow colour, was first identified in 1910 by Lampe and Milobedzka (Esatbeyoglu et al. 2012).

The rhizomes of *C. longa* have been used in Indian cuisine and possess a long history of use in Ayurvedic medicine for treatment of inflammatory conditions. Various pharmacological properties such as antitumor (Singh et al. 1996), antimicrobial (Negi et al. 1999), anti-inflammatory (Arora et al. 1971), antioxidant (Srinivas et al. 1992), anti-apoptotic (Chan and Wu 2004) and acetylcholinesterase inhibitory activities (Ahmed and Gilani 2009) were described for this plant.

Turmeric also inhibits NF-κB activation by receptor activator of nuclear factor kappa-B ligand (RANKL) which was correlated with the suppression of osteoclastogenesis (Kim et al. 2012). Moreover, it has been shown that the plant prevents inflammation through blockage of NF-κB in mucosa in dextran sodium sulphate (DSS)-induced chronic colitis (Deguchi et al. 2007) and inhibits immunostimulatory functions of dendritic cells by blocking MAPKs and NF-κB activation (Kim et al. 2005). So far, considerable data is available about *C. longa* anti-inflammatory properties, but little and conflicting information has been reported about the effect of *C. longa* on intestinal motility. In a clinical trial, turmeric therapy activated bowel motility and carbohydrate colonic fermentation (Shimouchi et al. 2009). However, the results of an animal study showed that curcumin decreases intestinal motility (Kumar et al. 2010) and curcuminoids produced a relaxant effect on smooth muscle in isolated guinea-pig ileum and rat uterus via receptor-dependent and independent mechanisms (Itthipanichpong et al. 2003).

The present study examined the relaxant effect of hydro-ethanol extract of *C. longa* on tracheal smooth muscle and the possible mechanism(s) underlying this effect.

## Materials and methods

### Plant and extract

*C. longa* rhizomes were purchased from an herbal store in Mashhad, Khorasan Razavi province, Iran in May 2015 and identified by Mr. Joharghi, a botanist form School of Agriculture, Ferdowsi University of Mashhad, Mashhad, Iran.

*C. longa* rhizomes were carefully cleaned, dried in shadow and grounded into powder. Next, powdered rhizome (100 g) was soaked in a 70% hydro-ethanol solution for 72 h, with occasional shaking and stirring at 37 °C. The mixture was then filtered and the resulting solution was concentrated under reduced pressure at 45 °C in an Eyela (Heidolph, Germany) rotary evaporator (Salama et al. 2013). The yield of the extract was 13% and it was stored at −70 °C until use.

### Characteristics of curcumin in the extract

**Figure 1.**
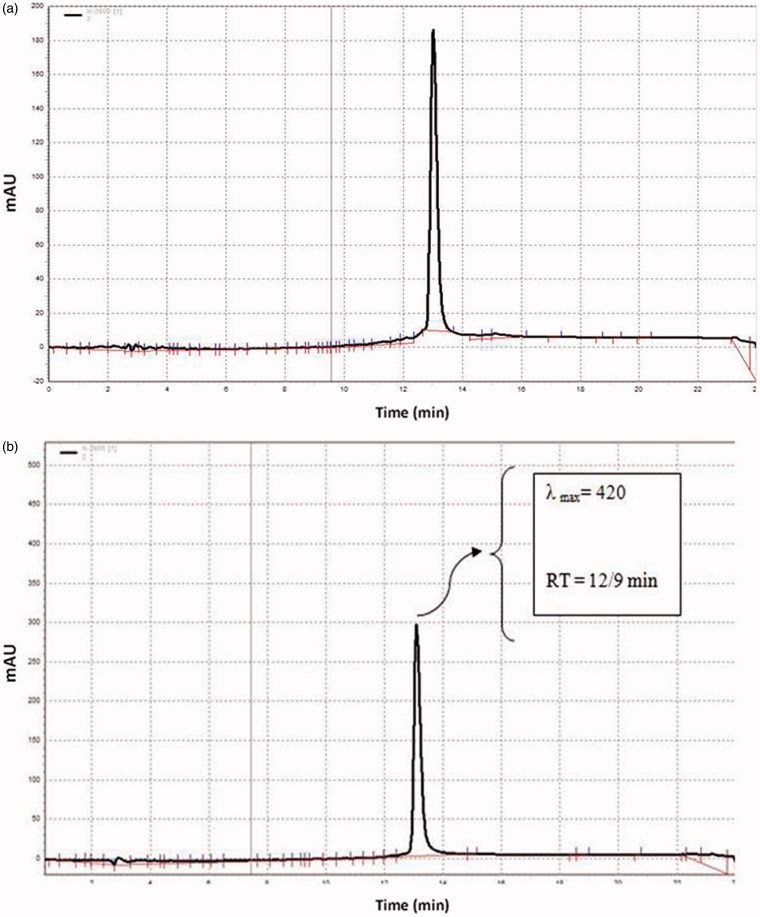
RP-HPLC of (a) the extract of *C. longa* and (b) curcumin.

The chromatograms of the extract and curcumin were recorded at 420 nm (the specific wavelength of curcumin). The other conditions of HPLC analysis included: Flow rate 1 mL/min; column type C18; column size 250 × 4.6 mm; and particle size 5 µm. The content of curcumin in the ethanol extract was 9.4%. No other impurities were observed in the extract at 420 nm (Figure 1).

### Animals

The experiments were performed using 84 male Wistar rats (weighing 200–250 g) obtained from the animal house of School of Medicine, Mashhad University of Medical Sciences, Mashhad, Iran. The animals were kept at 22 ± 2 °C with 12 h light/dark cycles and they had free access to standard diet and tap water, *ad libitum*. The study was approved by the Ethics Committee of Mashhad University of Medical Sciences (# 930658).

### Preparation of tissues

Rats were sacrificed and the chest was opened to obtain the trachea. The trachea was dissected and excess connective tissue and fat were removed. Then, the trachea was cut into rings of 3–4 mm width each having about four cartilages for the formation of tracheal ring. Each tracheal ring was hung between two Nichrome hooks inserted into the lumen, and placed in a 10 mL organ bath containing Krebs-Henseliet solution (KHS; composition (mM): NaCl 120, KCl 4.72, KH_2_PO_4_ 1.2, MgSO_4_·7H_2_O 0.5, CaCl_2_·2H_2_O 2.5, NaHCO_3_ 25 and dextrose 11). This solution was maintained at 37 ± 0.5 °C and constantly bubbled with 5% CO_2_-95% O_2_. Tissue was suspended under isotonic tension of 1 g and allowed to equilibrate for at least 1 h while it was being washed with KHS solution every 15 min. In all experiments, contraction responses were measured using an isotonic transducer (MLT0202, AD Instruments, Australia) which was connected to a power lab system (PowerLab 8/30, ML870, AD Instruments, Australia).

### Protocol

Concentration-response curves were plotted by treatment of pre-contracted tracheal smooth muscle with cumulative concentrations of *C. longa* extract or theophylline as positive control, at 5 min intervals. To create cumulative concentration of the extract, after adding the first concentration (6.25 mg/mL), at 5 min intervals, 6.25, 12.5 and 25 mg/mL solutions were added to organ bath to obtain 6.25, 12.5, 25, 50 mg/mL. For theophylline, after the first concentration, three 0.2 mM solutions were added to organ bath at 5 min intervals to create 0.2, 0.4, 0.6 and 0.8 mM concentrations. The effect of treatment with 1 mL saline (as negative control) on pre-contracted tracheal smooth muscle was also evaluated. To plot the concentration-response curve in each experiment, the relaxation% due to each concentration of the extract in proportion to the maximum contraction due to contractile agent (methacholine or KCl) was plotted against the extract concentration. The effective concentration of the extract causing 50% of maximum response (EC_50_) was also calculated as previously described by Sigmoid Emax model using GraphPad Prism version 6.00 for Windows (GraphPad program, GraphPad, San Diego, CA, USA) (Boskabady, Rahbardar, et al. 2010).

### Experimental groups

**Figure 2.**
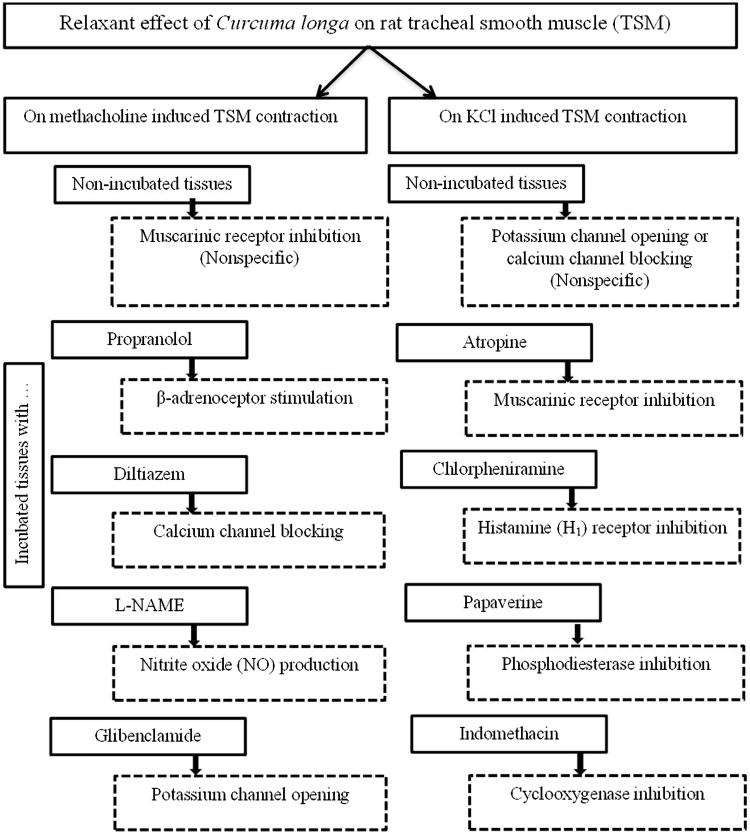
A diagram of the protocol of the study and the methods of evaluating of different mechanisms of the relaxant of effect of *Curcuma longa* the extract on tracheal smooth muscle.

The relaxant effect of the extract of *C. longa* was studied on tracheal smooth muscle contracted by methacholine or KCl for 5 min on non-incubated tissues or tissues incubated with different substance for 10 min as follows (Figure 2):

- Tracheal smooth muscle contracted by methacholine (Sigma Chemical Ltd, UK) on:
- Non-incubated tissues (*n* = 8) or tissues incubated with:
- 1 μM propranolol (Sigma Chemical Ltd UK), (*n* = 8)
- 5 μM diltiazem (Sigma Chemical Ltd UK), (*n* = 8)
- 300 μM L-NAME (Sigma Chemical Ltd UK), (*n* = 7)
- 1 μM glibenclamide (Sigma Chemical Ltd UK), (*n* = 7)
- Tracheal smooth muscle contracted by 60 mM KCl (Merk, Germany) on:
- Non-incubated tissues (*n* = 9) or tissues incubated with:
- 1 μM atropine (Sigma Chemical Ltd UK), (*n* = 7)
- 1 μM chlorpheniramine (Sigma Chemical Ltd UK), (*n* = 8)
- 1 μM indomethacin (Sigma Chemical Ltd UK), (*n* = 8)
- 100 μM papaverine (Sigma Chemical Ltd UK), (*n* = 8)

The above groups were done in random order in different tissues with a 60 min rest between the two experiments while tissues were being washed with KHS solution every 15 min. The relaxant effect of the extract on tissues incubated with the above agents was examined as the same as in non-incubated condition by using cumulative concentration of the extract, 10 min after adding the incubating agent to organ bath.

The effect of theophylline (positive control) was only examined on non-incubated tissues (*n* = 6 for each group). In addition, the effect of 1 mL saline (negative control) was examined at the beginning of each experiment.

## Data analysis

All values are presented as mean ± SEM. The data were analysed using one-way analysis of variance (ANOVA) followed by Tukey’s Multiple Comparison test. A *p* < 0.05 was considered statistically significant.

## Results

### The relaxant effect of *C. longa* extract onmethacholine-induced contraction in non-incubated tracheal smooth muscle

**Figure 3.**
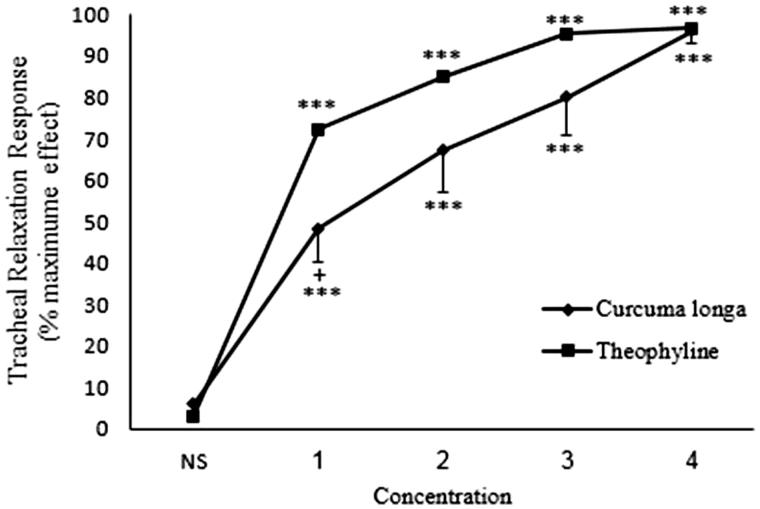
Concentration-response relaxant effect (mean ± SEM) of *C. longa* (*n* = 8) and theophylline (*n* = 6) on methacholine (10 μM) -induced contraction of rat tracheal smooth muscle in non-incubated tissues. ***: *p* < 0.001 compared to saline. +: *p* < 0.05 compared to the effect of theophylline. Statistical comparison was performed using ANOVA with Tukey Kramer post-test.

In non-incubated tissues contracted by methacholine, all concentrations of theophylline and *C. longa* extract showed significant and concentration-dependent relaxant effects compared to saline (*p* < 0.001 for all concentrations) (Figure 3). There were significant correlations between the relaxant effect and different concentrations of theophylline (*r* = 0.615, *p* < 0.001) and *C. longa* extract (*r* = 0.804, *p* < 0.001) in non-incubated tissues contracted by methacholine (Table 1). The relaxant effect of only the lowest concentration of the extract was significantly lower than that of theophylline (*p* < 0.05) (Figure 3).

**Table 1.** Relationship between the relaxant effect of the extract of *C. longa* and theophylline with their concentrations in different experimental groups.

Contractile agents	Studied agents	Conditions	*R*	*p-*value
Methacholine	*C. longa*	Non-Inc	0.804	*p* < 0.001
		Propranolol-Inc	0.623	*p* < 0.001
		Glibenclamide-Inc	0.785	*p* < 0.001
		Diltiazem-Inc	0.699	*p* < 0.001
		L-NAME-Inc	0.854	*p* < 0.001
	Theophylline	Non-Inc	0.615	*p* < 0.001
KCl	*C. longa*	Non-Inc	0.573	*p* < 0.001
		Atropine-Inc	0.652	*p* < 0.001
		Chlorpheniramine-Inc	0.736	*p* < 0.001
		Indomethacin-Inc	0.868	*p* < 0.001
		Papaverine-Inc	0.815	*p* < 0.001
	Theophylline	Non-Inc	0.823	*p* < 0.001

Inc: incubated tissues.

### The relaxant effect of *C. longa* extract onmethacholine-induced contraction in tissues incubated with propranolol

**Figure 4.**
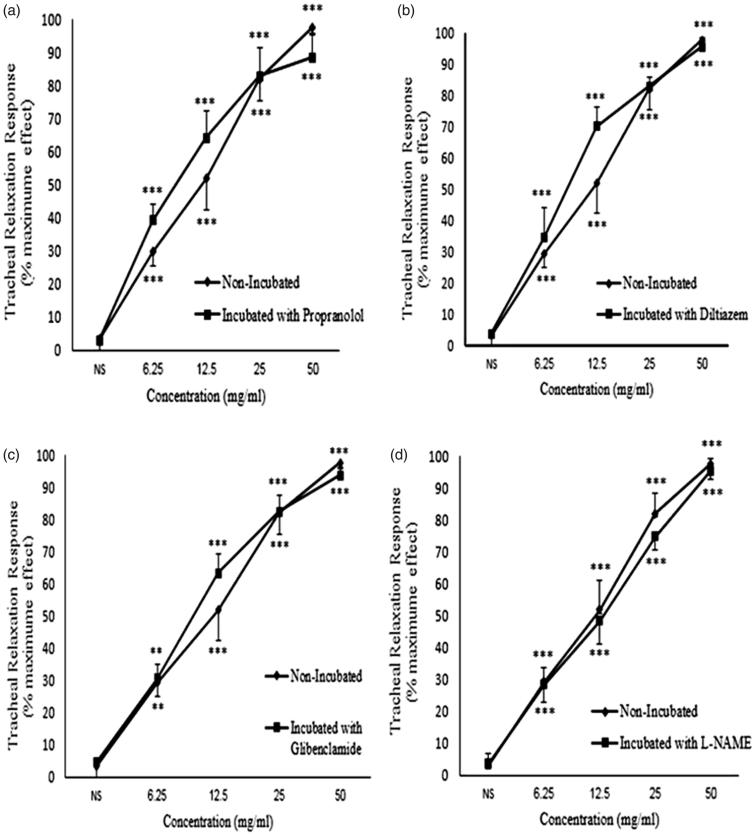
Concentration-response relaxant effect (mean ± SEM) of *C. longa* on methacholine (10 μM) -induced contraction of rat tracheal smooth muscle in non-incubated (*n* = 8) and incubated tissues with (a) propranolol (1 μM, *n* = 8), (b) diltiazem (5 μM, *n* = 8), (c) glibenclamide (1 μM, *n* = 7) and (d) L-NAME (300 μM, *n* = 7). ***: *p* < 0.001 compared to saline. There was no significant difference in the effect of the extract between non-incubated and incubated tissues with different agents. Statistical comparison was performed using ANOVA with Tukey Kramer post-test.

In tracheal smooth muscle incubated with propranolol, *C. longa* extract showed significant and concentration-dependent relaxant effects (*p* < 0.001 for all concentrations) (Figure 4(a)). The relaxant effects of *C. longa* extract in tissues incubated with propranolol significantly correlated with its concentrations (*r* = 0.623, *p* < 0.001; Table 1). There was no significant difference in the relaxant effects of *C. longa* extract between non-incubated tissue and tissue incubated with propranolol (Figure 4(a)).

### The relaxant effect of *C. longa* extract on methacholine-induced contraction in tissues incubated with diltiazem

*C. longa* extract also showed significant and concentration-dependent relaxant effects on tracheal smooth muscle incubated with diltiazem (*p* < 0.001 for all concentrations) (Figure 4(b)). A significant correlation was seen between the relaxant effect of *C. longa* extract and its concentrations in tissues incubated with diltiazem (*r* = 0.699, *p* < 0.001; Table 1). No significant difference was observed in the relaxant effects of *C. longa* extract between non-incubated tissue and tissue incubated with diltiazem (Figure 4(b)).

### The relaxant effect of *C. longa* extract onmethacholine-induced contraction in tissues incubated with glibenclamide

Significant and concentration-dependent relaxant effects were induced by various concentrations of *C. longa* extract in tracheal smooth muscle incubated with glibenclamide (*p* < 0.001 for all concentrations), (Figure 4(c)). There was a significant correlation between the relaxant effect of *C. longa* extract in tissues incubated with glibenclamide and the extract concentrations (*r* = 0.785, *p* < 0.001; Table 1). The relaxant effects of various concentrations of *C. longa* extract were not significantly different when comparing non-incubated tissue with tissue incubated with glibenclamide (Figure 4(c)).

### The relaxant effect of *C. longa* extract on methacholine-induced contraction in tissues incubated with L-NAME

In tracheal smooth muscle incubated with L-NAME, *C. longa* extract also showed significant and concentration-dependent relaxant effects (*p* < 0.001 for all concentrations) (Figure 4(d)). The relaxant effect of *C. longa* extract in tissues incubated with L-NAME significantly correlated with the extract concentrations (*r* = 0.854, *p* < 0.001; Table 1). There was no significant difference in the relaxant effects of *C. longa* extract between non-incubated tissue and tissue incubated with L-NAME (Figure 4(d)).

### Comparison of the relaxant effect of *C. longa* extract on methacholine-induced contraction in tissues incubated with different substances

The relaxant effects of various concentrations of the plant extract on methacholine-induced contraction were not significantly different among tracheal smooth muscle incubated with propranolol, diltiazem, glibenclamide or L-NAME (Table 2).

**Table 2.** Comparison of the relaxant effect of the extract of *C. longa* (percentage change in proportion to the maximum contraction) in different incubated tracheal smooth muscles contracted by 10 µM methacholine, (*n* = 9).

Concentration (mg/ml)				Incubating substance
50	25	12.5	6.25	
88.52 ± 7.02	82.79 ± 8.82	64.13 ± 8.09	39.22 ± 4.92	Propranolol
95.45 ± 2.92	82.81 ± 3.06	70.11 ± 6.01	34.42 ± 9.8	Diltiazem
95.48 ± 2.77	74.78 ± 4.03	48.45 ± 7.41	28.49 ± 5.57	L-NAME
93.77 ± 2.66	82.34 ± 5.32	63.44 ± 6.06	30.68 ± 4.18	Glibenclamide

Data were presented as mean ± SEM. There was not any significant difference in the relaxant effect of the extract between various incubated tissues.

### Comparison of EC_50_ values of *C. longa*-induced relaxation in tracheal smooth muscle contracted by methacholine

**Figure 5.**
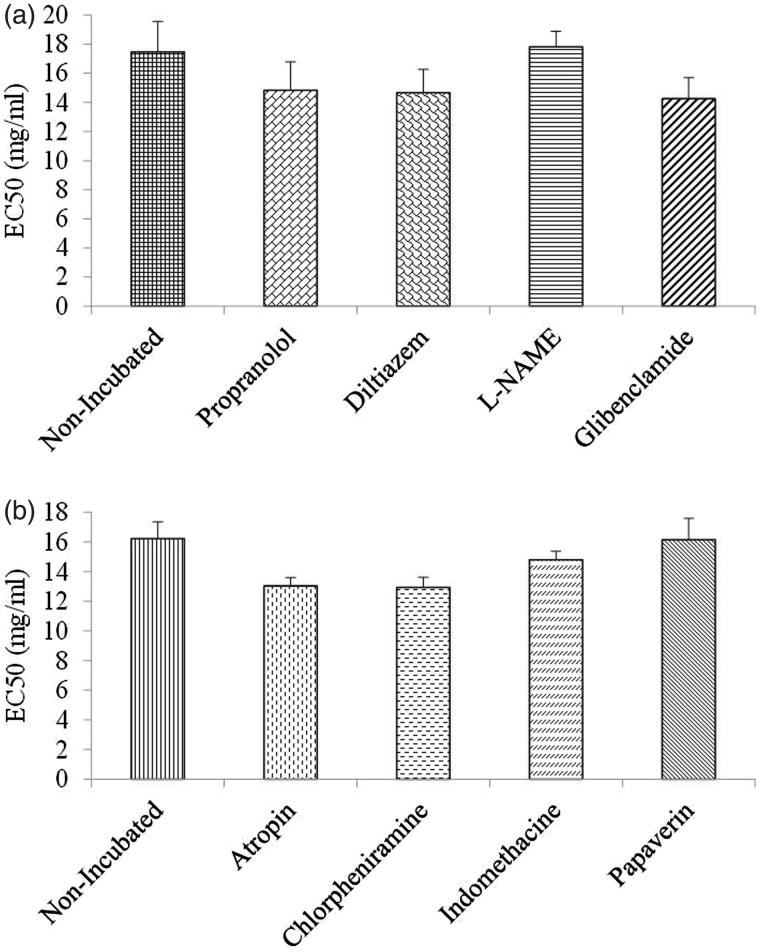
EC_50_ values of *C. longa* extract induce relaxation obtained on contracted tracheal smooth muscles of rat with (a) 10 μM methacholine, in non-incubated and incubated tissues with propranolol, diltiazem, L-NAME and glibenclamide (*n* = 8 for non-incubated and incubated tissues with propranolol and diltiazem, *n* = 7 for incubated tissues with L-NAME and glibenclamide) and (b) 60 mM KCl, in non-incubated and incubated tissues with atropine, chlorpheniramine, indomethacin and papaverine (*n* = 9 for non-incubated, *n* = 7 for incubated tissues with atropine and *n* = 8 for incubated tissues with chlorpheniramine, indomethacin and papaverine).There was no significant difference in EC_50_ values between non-incubated and incubated tissues with various agents. Statistical comparison was performed using ANOVA with Tukey Kramer post-test.

There was no significant difference among EC_50_ values of the extract in non-incubated tissue (17.46 ± 2.09) and tissues incubated with propranolol (14.83 ± 1.96), diltiazem (14.66 ± 1.61), glibenclamide (14.25 ± 1.44) or L-NAME (17.80 ± 1.07) (Figure 5(a)).

### The relaxant effect of *C. longa* extract on KCl-induced contraction in non-incubated rat tracheal smooth muscle

**Figure 6.**
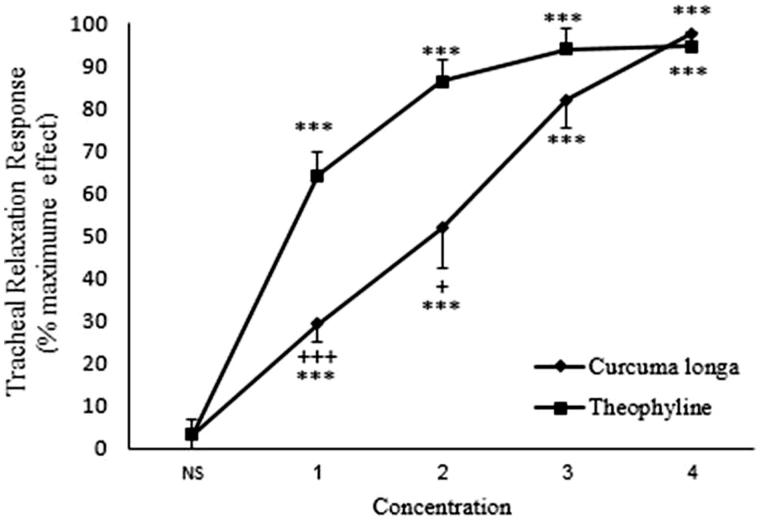
Concentration-response relaxant effect (mean ± SEM) of *C. longa* (*n* = 8) and theophylline (*n* = 6) on KCl (60 mM) -induced contraction of rat tracheal smooth muscle in non-incubated (*n* = 9) tissues. ***: *p* < 0.001 compared to saline. +++: *p* < 0.001, +: *p* < 0.05 compared to the effect of theophylline. Statistical comparison was performed using ANOVA with Tukey Kramer post-test.

In non-incubated tissues contracted by KCl, all concentrations of theophylline and *C. longa* extract showed significant and concentration-dependent relaxant effects compared to saline (*p* < 0.001 for all concentrations) (Figure 6). There were significant correlations between the relaxant effect and different concentrations of theophylline (*r* = 0.823, *p* < 0.001) and *C. longa* extract (*r* = 0.573, *p* < 0.001) in non-incubated tissues contracted by KCl (Table 1). The relaxant effect of the two lower concentrations of the extract was significantly lower than those of theophylline (*p* < 0.001 and *p* < 0.05 for 6.25 and 12.5 mg/mL concentrations, respectively) (Figure 6).

### The relaxant effect of *C. longa* extract on KCl-induced contraction in tissues incubated with atropine

**Figure 7.**
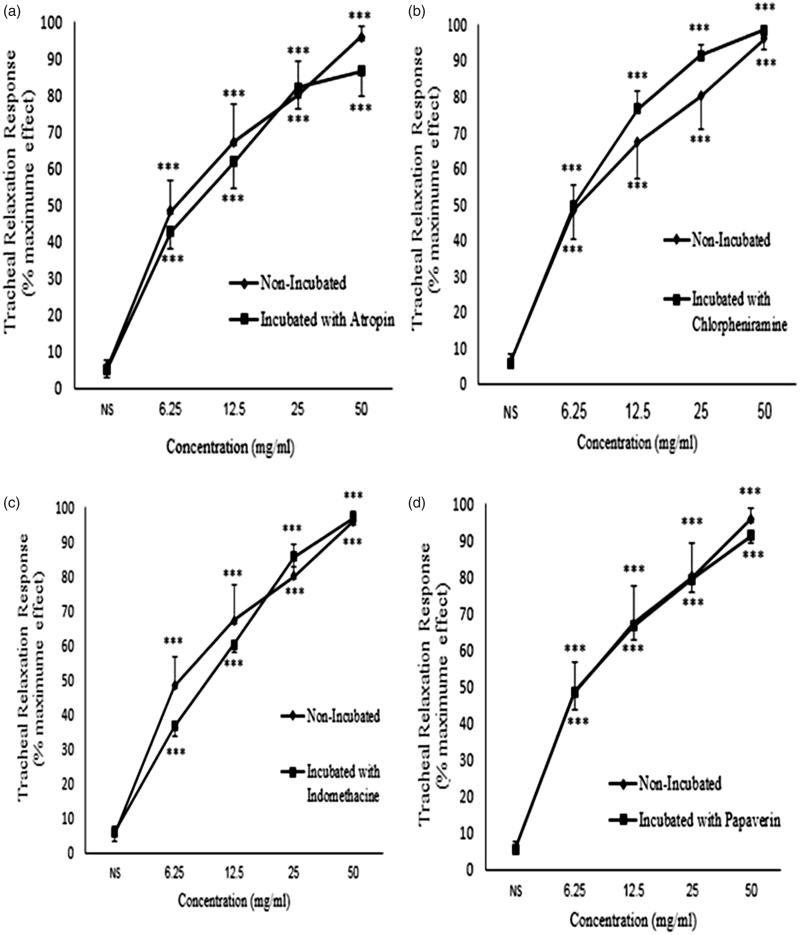
Concentration-response relaxant effect (mean ± SEM) of *C. longa* on KCl (60 mM) -induced contraction of rat tracheal smooth muscle in non-incubated (*n* = 9) and incubated tissues with (a) atropine (1 μM, *n* = 7), (b) chlorpheniramine (1 μM, *n* = 8), (c) indomethacin (1 μM, *n* = 8), (d) papaverine (100 μM, *n* = 8). ***: *p* < 0.001 compared to saline. There was no significant difference in the effect of the extract between non-incubated and incubated tissues. Statistical comparison was performed using ANOVA with Tukey Kramer post-test.

In tracheal smooth muscle incubated with atropine, *C. longa* extract showed significant and concentration-dependent relaxant effects (*p* < 0.001 for all concentrations) (Figure 7(a)). The relaxant effect of *C. longa* extract in tissues incubated with atropine significantly correlated with the extract concentrations (*r* = 0.652, *p* < 0.001; Table 1). There were no significant differences in the relaxant effects of *C. longa* extract between non-incubated tissue and tissue incubated with atropine (Figure 7(a)).

### The relaxant effect of *C. longa* extract on KCl-induced contraction in tissues incubated with chlorpheniramine

*C. longa* extract showed significant and concentration-dependent relaxant effects in tracheal smooth muscle incubated with chlorpheniramine (*p* < 0.001 for all concentrations) (Figure 7(b)). There was a significant correlation between the relaxant effect of *C. longa* extract in tissues incubated with chlorpheniramine and the extract concentrations (*r* = 0.736, *p* < 0.001; Table 1). However, there were no significant differences in the relaxant effects of *C. longa* extract between non-incubated tissue and tissue incubated with chlorpheniramine.

### The relaxant effect of *C. longa* extract on KCl-induced contraction in tissues incubated with indomethacin

Significant and concentration-dependent relaxant effects were observed for *C. longa* extract in tracheal smooth muscle incubated with indomethacin (*p* < 0.001 for all concentrations) (Figure 7(c)). A significant correlation was seen between the relaxant effects of *C. longa* extract and its concentrations in tissues incubated with indomethacin (*r* = 0.868, *p* < 0.001; Table 1). No significant differences were seen for the relaxant effects of various concentrations of *C. longa* extract between non-incubated tissue and tissue incubated with indomethacin (Figure 7(c)).

### The relaxant effect of *C. longa* extract on KCl-induced contraction in tissues incubated with papaverine

Significant and concentration-dependent relaxant effects of *C. longa* extract were observed in tracheal smooth muscle incubated with papaverine (*p* < 0.001 for all concentrations) (Figure 7(d)). The relaxant effects of the extract of *C. longa* showed a significant correlation with its concentrations in tissues incubated with papaverine (*r* = 0.815, *p* < 0.001, Table 1). The relaxant effects of *C. longa* extract were not significantly different between non-incubated tissue and tissue incubated with papaverine (Figure 7(d)).

### Comparison of the relaxant effect of *C. longa* on KCl-induced contraction in tissues incubated with different substances

There were no significant differences in the relaxant effects of various concentrations of the extract of the plant on KCl-induced muscle contraction among tracheal smooth muscle tissues incubated with different agents (atropine, chlorpheniramine, indomethacin and papaverine) (Table 3).

**Table 3.** Comparison of the relaxant effect of the extract of *C. longa* (percentage change in proportion to the maximum contraction) in different incubated tracheal smooth muscles contracted by 60 mM KCl, (*n* = 9).

Concentration (mg/ml)				Incubating substance
50	25	12.5	6.25	
86.57 ± 7.03	82.1 ± 5.98	61.78 ± 7.18	42.74 ± 4.46	Atropine
98.47 ± 0.75	91.31 ± 2.89	75.46 ± 4.9	49.6 ± 5.92	Chlorpheniramine
96.99 ± 2.13	85.65 ± 3.07	60.28 ± 2.41	36.72 ± 3.09	Indomethacin
91.25 ± 2.12	79.48 ± 3.83	66.6 ± 33.82	48.63 ± 5.02	Papaverine

Data were presented as mean ± SEM. There was not any significant difference in the relaxant effect of the extract between various incubated tissues.

### Comparison of EC_50_ values of *C. longa*-induced relaxation in tracheal smooth muscle contracted by KCl

The EC_50_ values of the extract obtained in non-incubated tissue (16.22 ± 0.62) and those of tissue incubated with atropine (13.03 ± 0.55), chlorpheniramine (12.94 ± 0.68), indomethacin (14.80 ± 0.57) and papaverine (16.16 ± 1.42) were not significantly different (Figure 5(b)).

### Comparison of the relaxant effect of *C. longa* between non-incubated tissues contracted by KCl and methacholine

**Figure 8.**
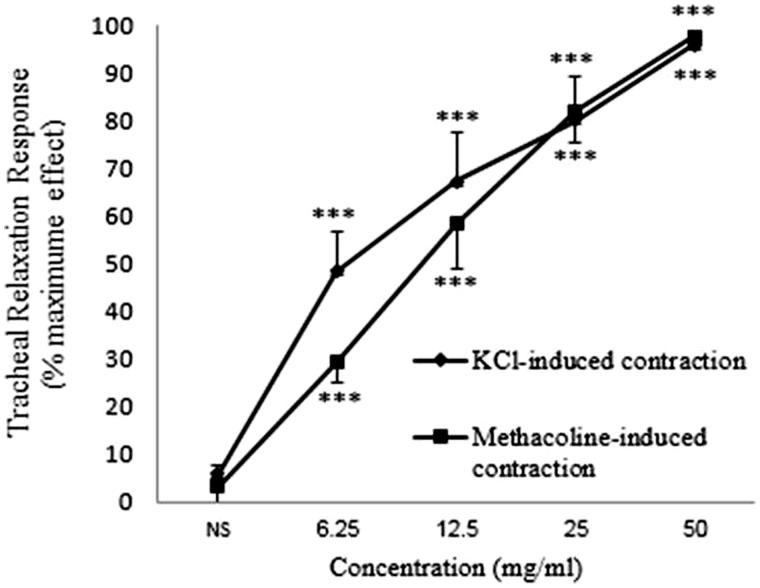
Concentration-response relaxant effect (mean ± SEM) of *C. longa* on KCl (60 mM) and methacholine (10 μM) -induced contraction of tracheal smooth muscle of rat in non-incubated tissues (*n* = 9 for each group). ***: *p* < 0.001 compared to saline. There was no significant difference in the relaxant effect of the extract between KCl and methacholine-induced contraction of tracheal smooth muscle. Statistical comparison of the effect of each concentration between two groups was performed using unpaired *t*-test.

The relaxant effects of various concentrations of *C. longa* extract in tracheal smooth muscle contracted by methacholine were not significantly different from those seen in KCl-contracted tissues (Figure 8).

## Discussion

In the present study, the relaxant effect of the hydro-ethanol extract of *C. longa* on tracheal smooth muscle was examined to access of the possibility of application of this plant as a bronchodilator drug for treatment of obstructive pulmonary diseases. The relaxant effect of the plant on methacholine and KCl-induced muscle contraction was examined in non-incubated and incubated tissues to understand the underlying mechanism(s).

The extract of *C. longa* showed a significant and concentration-dependent relaxant effect in non-incubated tracheal smooth muscle contracted with methacholine. There were no significant differences between the relaxant effects of the three higher concentrations of the extract and those of theophylline in this group. The results of this group indicated a relatively potent relaxant effect of the plant which was almost comparable to that of theophylline at studied concentrations.

In the next group, the relaxant effect of the plant was examined in tracheal smooth muscle incubated with propranolol and contracted with methacholine, to examine the contribution of β-adrenoceptor stimulatory effect of the plant to its smooth muscle relaxant effect. It is well known that β-adrenoceptor stimulators are one of the main tracheal smooth muscle relaxant (bronchodilator) drugs (Nelson 1995). In this group, the extract also showed significant and concentration-dependent relaxant effects on tracheal smooth muscle which were not significantly different from those of the extract in non-incubated tissues. If the relaxant effect of the plant was due to its stimulatory effect on β-adrenoceptor, its relaxant effect would be inhibited or at least decreased in tissues incubated with propranolol (Koushyar et al. 2013; Boskabady, Kaveh, et al. 2010); Therefore, the results of this group suggest that the relaxant effect of *C. longa* on tracheal smooth muscle is not due to its β-adrenoceptor stimulatory property. The effect of *C. longa* on carbachol, histamine, K^+^-induced contraction in isolated ileum was investigated in mice and findings showed that spasmolytic effect of *C. longa* on K^+^-induced contraction was mediated through L type calcium channels. The competitive antagonism of the extract on cholinergic, histaminergic and serotoninergic receptors does not contribute to its relaxant effect (Micucci et al. 2013) which exactly supports the results of the present study.

The relaxant effects of *C. longa* were also examined in tracheal smooth muscle incubated with diltiazem and contracted with methacholine to evaluate the contribution of calcium channel blockade to the relaxant effect of the plant. Calcium channel blocking agents can also relax tracheal smooth muscle (Sonna et al. 1996). The plant extract also showed a significant and concentration-dependent relaxant effect in this group. There was no significant difference in the relaxant effect of different concentrations of the extract between non-incubated tissues and tissues incubated with diltiazem. These results showed that *C. longa* has no inhibitory effect on calcium channels.

In order to examine the effect of the plant on potassium channels, the relaxant effect of the extract was examined on tracheal smooth muscle incubated with glibenclamide and contracted with methacholine. It is well known that glibenclamide blocks potassium channels (Teramoto et al. 2004). The relaxant effects of potassium channel openers on tracheal smooth muscle have also been documented (Miura et al. 1992). The relaxant effect of the extract on tracheal smooth muscle incubated with glibenclamide was not significantly different from that of the plant in non-incubated tissues. Therefore, the relaxant effect of the plant is not due to its potassium channel opening activity.

Moreover, the relaxant effect of *C. longa* was assessed on tracheal smooth muscle incubated with L-NAME to examine the role of nitrite oxide (NO) production on the relaxant effect of the plant because the relaxant effect of NO on tracheal smooth muscle was shown previously (Insuela et al. 2015). No differences were seen in the relaxant effect of the extract between non-incubated and tissues incubated with L-NAME, indicating that the effect of the plant does not involve NO production and this mechanism is not responsible for the relaxant effect of *C. longa* on tracheal smooth muscle.

In non-incubated tracheal smooth muscle contracted by KCl, the extract of *C. longa* showed significant and concentration-dependent relaxant effects. The relaxant effect of the two higher concentrations of the extract on KCl-induced contraction of tracheal smooth muscle was not significantly different from that of theophylline. |In tracheal smooth muscle contracted by KCl, the plant showed significant relaxant effect which was comparable to that of theophylline at studied concentration. There was no significant difference in the relaxant effect of different extract concentrations between tracheal muscle contracted by KCl and tracheal muscle contracted by methacholine.

Similar relaxant effect of the extract was observed in both KCl and methacholine-contracted smooth muscle may suggest that the relaxant effect of the extract is mainly mediated via its inhibitory effect on calcium channels and/or opening effect on potassium channels (Buckle et al. 1993; Laurent et al. 1993). However, these results are in contrast with those observed in tissues incubated with diltiazem and glibenclamide. If calcium channel inhibitory or potassium channel opening effect of the plant contributes to its relaxant effect, this effect should be inhibited or at least reduced in tissues incubated with diltiazem and glibenclamide. Therefore, the relaxant effect observed for *C. longa* extract in tracheal smooth muscle contracted by KCl, is a non-specific effect.

The relaxant effect of the extract was also tested in tissues incubated with atropine and contracted with KCl to examine the role of muscarinic receptors in the relaxant effect of *C. longa* extract. The relaxant effects of muscarinic inhibitors were shown previously (Lronards et al. 1992) and these agents are used as a class of bronchodilators (Spina 2014). The results of this group showed a significant and concentration-dependent relaxant effect for the extract which was not significantly different from that of the plant in non-incubate tissues contracted with KCl. The findings of this group showed that the plant had no inhibitory effect on muscarinic receptors and therefore, this mechanism is not responsible for the relaxant effect of *C. longa* on tracheal smooth muscle.

In tracheal smooth muscle incubated with chlorpheniramine, the relaxant effect of the plant was studied to examine the role of histamine (H_1_) receptors in the relaxant property of the extract. The relaxant effect of histamine (H_1_) receptor blocking drugs on tracheal smooth muscle (bronchodilator effect) was previously reported (Popa 1977). The non-significant difference in the relaxant effect of the extract observed between non-incubated tissues and tissues incubated with chlorpheniramine, indicated that *C. longa* had no inhibitory effect on (H_1_) receptor of tracheal smooth muscle.

Furthermore, the relaxant effect of the plant in tracheal smooth muscle incubated with papaverine was also studied to examine the contribution of phosphodiesterase inhibitory mechanism to the relaxant effect of the plant. In previous studies, the relaxant effect of phosphodiesterase inhibitors was demonstrated (Bardou et al. 2002). However, the results of this group showed non-significant differences in the relaxant effect of *C. longa* between non-incubated tissues and tissues incubated with papaverine. These findings suggest that phosphodiesterase inhibition does not contribute to the relaxant effect of the plant on tracheal smooth muscle.

Finally, the relaxant effect of the extract was examined in tissues incubated with indomethacin. In this group, the role of anti-inflammatory property of the plant in its relaxant effect was evaluated. In fact, the anti-inflammatory effect of *C. longa* was shown previously (Kamarudin et al. 2011). The results of this group also showed that the relaxant effect of the plant in non-incubated tissues was not significantly different from that seen in tissues incubated with indomethacin. The results of this group indicated that the anti-inflammatory property of the plant does not affect its tracheal smooth muscle relaxant activity.

The results also showed no significant differences in the relaxant effect of *C. longa* between tissues incubated with different agents, in both methacholine and KCl-induced muscle contraction. The relaxant effect of the plant in non-incubated tissues was also not significantly different between methacholine and KCl-contracted muscle tissues. The EC_50_ values of the plant-induced relaxation were also not significantly different between non-incubated tissues and tracheal smooth muscle incubated with different agents in both methacholine and KCl-induced muscle contraction. These results also confirmed that the studied mechanisms do not contribute to the relaxant effect of *C. longa* on tracheal smooth muscle.

The relaxant effects of *C. longa* in all experimental groups were concentration-dependent and there were significant correlations between the relaxant effects and the concentrations of the extract. The relationships between the relaxant effects of theophylline and its concentrations were significant in both methacholine and KCl-contracted muscle tissues. The close correlation between the effect of the plant and its concentrations showed concentration-dependency of the relaxant effect of *C. longa* and supports its potent relaxation effect on tracheal smooth muscle. In fact, the relaxant effects of this plant on different types of smooth muscle were shown previously (Itthipanichpong et al. 2003; Kumar et al. 2010) which can support the findings of the present study.

Aaldini et al. (2012) showed that *C. longa* has a myorelaxant effect on mouse ileum and colon supporting the results of the present study. The possible mechanism of the relaxant effect of *C. longa* was determined by evaluation of its effect on carbachol-induced contraction in tissues incubated with atropine. The results suggested that a direct myorelaxant effect on the intestinal muscle layers and a non-competitive and reversible inhibition of the cholinergic agent were responsible for this effect (Aldini et al. 2012). The discrepancy between the findings of the above-mentioned study and the present experiment could be due to the differences between the cholinergic receptors in the ileum and colon and those of tracheal smooth muscles. Hydroalcoholic extract of a polyherbal formulation that consisted of *C. longa* and *Butea frondosa* (Fabaceae), showed muscle relaxant effects (Abid et al. 2011). The effect of *C. longa* on isolated rat superior mesenteric arteries also showed that *C. longa* has vasorelaxant effects mediated by stimulation of the forward mode of Na^+^-Ca^2+^exchanger (Adaramoye and Medeiros 2008) which also supports the findings of the present study showing the relaxant effect of the plant on smooth muscle. However, the role of stimulation of the forward mode of Na^+^-Ca^2+^exchanger in the relaxant effect of the plant on tracheal smooth muscle should be investigated in future studies.

The muscle relaxant effects of other species of curcuma such as the effect of hydroalcoholic extract of *Curcuma caesia* on isolated guinea pig trachea was shown and suggested to be mediated via modulation of calcium channels (Arulmozhi et al. 2006). The muscle relaxant effect of *C. caesia* extract was also suggested by another report (Karmakar et al. 2011). The effect of *Curcuma aeruginosa* on isolated uterus strips from rats was also demonstrated and suggested to be due to the presence of β2-pinene and some sesquiterpene lactones (Thaina et al. 2009). Therefore, the results of the present study as along with those of previous studies indicate smooth muscle relaxant effect of various Curcuma species.

In the present study, we recorded the potent relaxant effect of the extract of *C. longa* on tracheal smooth muscle which is a novel finding and has not been reported previously. However, the relaxant effect of the plant was not due to the examined mechanisms such as of β2-adrenergic receptors stimulation (Linden et al. 1993), histamine receptors inhibition (Popa et al. 1984), calcium channel blocking (Miyahara et al. 1993), potassium channel opening (Buckle et al. 1993), muscarinic receptors inhibition (Lronards et al. 1992), nitric oxide synthase (NOS) inhibition (Danser et al. 2000), phosphodiesterase inhibition (Shimizu et al. 2000), cyclooxygenase (COX) inhibition (Slattery et al. 2001) and ATP-sensitive potassium channels inhibition (Satoh et al. 1991) which are also novel results. The other possible mechanisms which may contribute to the relaxant effects of *C. longa* on tracheal smooth muscle including methylxanthine activity (Boskabady et al. 2004) and effect on non-adrenergic, non-cholinergic nervous system (NANC) (Li and Rand 1991), should be examined in future studies. Although the relaxant effect of the extracts of various medicinal plants and their possible mechanisms were studied and published (Shakeri and Boskabady 2015), the relaxant effect of various constituents of *C. longa* should be examined in further studies. In addition, the relaxant effect of the plants and their constituents and the possible mechanism of this effect should be examined in isolated cells from tracheal muscle in future.

## Conclusions

In conclusion, the results of the present study showed relatively potent relaxant effects of *C. longa* on tracheal smooth muscle of rats that were comparable to those of theophylline at studied concentrations. These results suggest that the plant extract could be used as a bronchodilator agent in the treatment of obstructive pulmonary diseases such as asthma and COPD. However, the findings showed that the relaxant effect of the extract was not due to its effect on β-adrenergic, muscarinic, or histamine (H_1_) receptors, calcium or potassium channels, NO production or phosphodiesterase activity. Therefore, in further studies, other mechanism(s) responsible for the relaxant effect of the plant on tracheal smooth muscle should be examined. In addition, clinical studies, evaluating the bronchodilatory effects of *C. longa* on obstructive pulmonary diseases are required for investigation of the possible clinical use of the plant.
